# On Cancer of the Pulmonary Artery

**Published:** 1856-01

**Authors:** A. Wernher

**Affiliations:** Giessen


### RECORD OF MEDICAL SCIENCE.

*On Cancer of the Pulmonary Artery.* By Professor A. Wernher, of Giessen.

The following case of cancer of the pulmonary artery presents a degree of interest in reference to the question of the mode of propagation of cancerous affections:—

*Case.*—C——, a servant aged 22, placed himself under M. Wernher's care on the 22nd of January, 1853. In his childhood he had suffered from several cerebral affections. His present disease was of three months' standing, but he said he had often, during the last year, experienced, especially in dancing, painful sensations in the thigh and knee, which he attributed to chills.

In the preceding autumn he had had attacks of angina and of swelling of the calf of the left leg. After these symptoms subsided, a small tumour, hard and but slightly painful, and to which he paid little attention, appeared at the inner side of the tibia. The tumour increasing rapidly, the patient made use of ointments and various other topical applications, but without success, for in the space of three months the swelling attained a considerable volume; two punctures had been made, but gave issue to nothing but blood; the tumour soon became painful, and the patient lost strength, and visibly emaciated.

On his admission into hospital the skin was hot and dry; the pulse small, from 120 to 140; his sleep was disturbed, being interrupted by pain; his appetite was good; the tongue was slightly loaded; there was some constipation; there was no enlargement of the liver; he had frequent attacks of epistaxis. From the time of his admission he had some cough, attended with dyspnœa and pain in a particular point at the left side. The left knee was bent almost at a right angle, and was nearly immoveable. The region below the condyles of the femur and the patella was occupied by a knobby tumour, the greatest circumference of which amounted to 99 centimetres (38.9 inches,) and which extended to the middle third of the tibia. The skin covering the tumor was tense and shining, covered with spots of a reddish yellow, and here and there exhibited varicose veins and networks of finer vessels; the temperature of this region was sensibly elevated. The tumour was elastic, hard in some parts, soft in others, particularly where the vessels were developed, but it presented no fluctuation. It was the seat of spontaneous shooting pains, particularly at night, and pressure also excited pain. The inguinal glands were scarcely swollen; no tumour was perceptible in the abdomen.

The tumour was considered to be a medullary fungus which should speedily prove fatal, and amputation was proposed, but was at first refused. However, the disease continuing to progress, the patient submitted to the operation, which was performed on the 7th of February, in the upper part of the thigh.

Some days before, the patient suddenly experienced a severe pain in the region of the heart, with dyspnœa and acceleration of the respiratory movements; subsequently, he had cough and bloody sputa, very rapid pulse, &c.

The frequency of the pulse diminished after the operation, as well as most of the symptoms; but the chest soon became more engaged, as was indicated by cough, dyspnœa, bloody, purulent, and fœtid sputa; miliary eruption, collapse, &c.

The patient died on the 24th of February.

The examination of the tumour showed, as had been diagnosed, a medullary fungus of the tibia.

The author gives a description of the elements of the tumour, with figures representing their form and disposition. The autopsy was performed seven hours after death.

The veins of the stump were perfectly healthy, and did not present the slightest trace of phlebitis.

The right lung exhibited in its middle part an enormous gangrenous abscess filled with a fœtid ichorous matter; another similar, but smaller, abscess was found in its inferior portion.

The rest of the parenchyma was healthy; but on incising it, little knotty cords, having the appearance of cancerous matter, were seen to protrude from the orifices of the vessels.

A preparation of the vessels of the lung, made with the greatest care, showed that these cords existed only in the pulmonary ramifications; nothing similar was found in the veins. The cords in question, which obstructed the bore of the vessels without adhering to their walls, were of a dull white color, nearly resembling that of half-boiled rice; knotty, gyrose, and transparent at the edges. Each of the stronger cords was composed of several others; these little cords were twisted, but separated easily without the aid of any instrument.

In the right lung all the ramifications of the artery, with few exceptions, were obstructed by these cancerous masses; there were fewer in the arteries of the left lung.

It was chiefly in the vicinity of the large abscess that the smallest vascular ramifications were completely stopped.

The points in which the cancerous matter had commenced to deposit could be observed; the smallest vessels which contained it were from a third to half a line in diameter; beyond that point they were gorged with blood; in this part they presented a sudden dilatation, and this, combined with their hardness, indicated the presence of the deposit.

The capillaries were entirely free; the same was true of the parenchyma of the lung beyond the vascular ramifications; no kind of tubercle was found in it.

Microscopic examination showed that the cords were composed of cells, analogous in their forms and dimensions to those of incipient cancer, being large and oval, with a single or double nucleus; on the surface of the cords were found caudate cells, arranged in several layers, and even forming an epithelium.

The same elements of cancer were found in the blood of the inferior cava, and in great quantity (oval or caudate, isolated or agglomerated.)

In no other part of the body, notwithstanding the most careful examination, could any purulent or cancerous deposit, or any abscess, be found.

This case is remarkable, chiefly because it shows the direct transfer of the cancerous matter, by the venous blood, to the very extremity of the vascular tree. No general cancerous infection existed; the cancerous cells, detached doubtless from the principal focus, had been conveyed with the blood and deposited in the branches of the pulmonary artery, and had there mechanically accumulated to such a degree as to obstruct these vessels. It is to this accumulation that we must attribute the symptoms of which the lung was the seat.

We must not, however, deduce from this particular case a general theory of the mode of transmission of cancerous affections, as the author appears disposed to do. It is certain that the elements of cancer must necessarily impart injurious qualities to the blood, and it is, in fact, this infected blood which, diffusing itself in all directions, finally produces new local affections.

Lastly, we would remark that the denomination of cancer of the pulmonary artery given by the author does not appear to us to be accurate; he himself states that the cancerous masses were free in the vascular tubes; these latter consequently were not diseased, and it does not appear from the details of the autopsy, that they were altered in their proper tissue. We are therefore justified in considering the cancerous masses contained in the vessels as being derived from the primary focus and transported directly towards the extremities of the venous tree, and it is thus that the author himself regards them.—*Dublin Med. Press. from Gazette Mêdicale de Paris,* October 27, 1855.

